# Surgical strategies for older patients with glioblastoma

**DOI:** 10.1007/s11060-021-03862-z

**Published:** 2021-10-09

**Authors:** Tanyeri Barak, Shaurey Vetsa, Arushii Nadar, Lan Jin, Trisha P. Gupte, Elena I. Fomchenko, Danielle F. Miyagishima, Kanat Yalcin, Sagar Vasandani, Evan Gorelick, Amy Y. Zhao, Joseph Antonios, Brianna Carusillo Theriault, Nathan Lifton, Neelan Marianayagam, Bulent Omay, Zeynep Erson Omay, Anita Huttner, Declan McGuone, Nicholas A. Blondin, Zachary Corbin, Robert K. Fulbright, Jennifer Moliterno

**Affiliations:** 1 Department of Neurosurgery, Yale School of Medicine, 15 York St., LLCI 810, CT 06520-8082 New Haven, USA; 2 Yale Brain Tumor Center, Smilow Cancer Hospital, CT New Haven, USA; 3 Department of Surgery, Yale School of Medicine, New Haven, USA; 4 Department of Environmental Health Sciences, Yale School of Public Health, New Haven, USA; 5 Department of Pathology, Yale School of Medicine, New Haven, USA; 6 Department of Neurology, Yale School of Medicine, New Haven, USA; 7 Department of Radiology and Biomedical Imaging, Yale School of Medicine, New Haven, USA; 8 Department of Genetics, Yale School of Medicine, New Haven, Connecticut, USA

**Keywords:** Glioblastoma, Surgical strategies, Neuronavigation, Intraoperative imaging

## Abstract

**Objective:**

While adjuvant treatment regimens have been modified for older patients with glioblastoma (GBM), surgical strategies have not been tailored.

**Methods:**

Clinical data of 48 consecutive patients aged 70 years or older, who underwent surgical resection for GBM with intraoperative ultrasonography (IoUS) alone or combination with intraoperative MRI (IoMRI) at Yale New Haven Hospital were retrospectively reviewed. Variables were analyzed, and comparative analyses were performed.

**Results:**

The addition of IoMRI was not superior to IoUS alone in terms of overall survival (OS) (P = 0.306), Karnofsky Performance Score (KPS) at postoperative 6 weeks (P = 0.704) or extent of resection (P = 0.263). Length of surgery (LOSx), however, was significantly longer (P = 0.0002) in the IoMRI group. LOSx (P = 0.015) and hospital stay (P = 0.025) were predictors of postoperative complications. Increased EOR (GTR or NTR) (P = 0.030), postoperative adjuvant treatment (P < 0.0001) and postoperative complications (P = 0.006) were predictive for OS. Patients with relatively lower preoperative KPS scores (<70) showed significant improvement at postoperative 6 weeks (P<0.0001). Patients with complications (P = 0.038) were more likely to have lower KPS at postoperative 6 weeks.

**Conclusions:**

Aggressive management with surgical resection should be considered in older patients with GBM, even those with relatively poor KPS. The use of ioMRI in this population does not appear to confer any measurable benefit over ioUS in experienced hands, but prolongs the length of surgery significantly, which is a preventable prognostic factor for impeding care.

**Supplementary Information:**

The online version contains supplementary material available at 10.1007/s11060-021-03862-z.

## Introduction

Glioblastoma (GBM) is the most common primary malignant brain tumor in adults. The incidence of GBM increases with advanced age, with a peak in the 75–84 age group [1]. Studies show that more than one fourth of GBM patients are older than 70 years of age at the time of diagnosis [2]. Recent studies centered on the use of radiation and chemotherapy in this older population have garnered results and recommendations to help specifically guide treatment of GBM [3–8] in this population, however strategies for surgical management have yet to be optimized.

Though there is compelling evidence that maximal safe resection and chemoradiation are associated with improved survival in younger GBM patients, many physicians have traditionally followed more conservative approaches when treating septuagenarians and octogenarians [9–11]. This paradigm, however, is recently challenged by a growing body of evidence demonstrating that elderly patients benefit from maximal safe resection and chemoradiation [3, 4, 5, 6, 7, 8, 12, 13, 14, 15, 16, 17, 18, 19, 20]. Indeed, improved survival with increased EOR in older GBM patients occurs in patients without postoperative complications [21] and prolonged surgery is an independent predictor of complications in elderly patients undergoing craniotomies [22–29].

More sophisticated intraoperative image guidance techniques have been used more routinely in GBM surgery to achieve maximal EOR. Though the use of intraoperative MRI (IoMRI) may now be considered the “gold standard”, it prolongs surgical time [30]. On the other hand, IoUS remains a reliable, real-time imaging tool, which improves surgical resection [31–35], while reducing the length of surgery (LOSx) compared with IoMRI. Surgeon expertise with its interpretation, however, is important for its success. Given the notion that increased surgical time could potentially be associated with postoperative complications, we sought to determine whether there were indeed any differences in outcome with these intraoperative image guidance techniques in older patients with GBM.

## Methods

### Clinical data acquisition

This study was approved by the Yale School of Medicine Institutional Review Board. Forty-eight consecutive patients, age 70 years and older, who underwent surgical resection by the senior author for histologically confirmed GBM between November 2015 and January 2021 were included. Demographic and clinical variables were retrospectively collected. All patients were followed until death or through submission of this manuscript with none lost during follow-up. Patients not meeting the aforementioned criteria and/or those who underwent biopsy were excluded.

### Functional assessment

Functional assessment was done using the Karnofsky Performance Scale (KPS) score, as determined by retrospective review of detailed medical records. Preoperative KPS was determined at the time of admission, while postoperative KPS was assessed at approximately 6 weeks follow-up. Functional improvement was determined based on the difference. To further evaluate the clinical improvement of patients, we also used The Neurologic Assessment in Neuro-Oncology (NANO) scale [36, 37].

### Tumor location, volume and extent of resection

Tumor volume was calculated using the modified ellipsoidal formula, *V* = (4/3) × π × (*D*_1_/2) × (*D*_2_/2) × (*D*_3_/2) based on the maximum tumor diameters in perpendicular dimensions. All patients underwent contrast enhanced T1-weighted MRI before surgery and within 48 h after surgery when possible and were used to determine the amount of tumor removed. All surgeries were performed by the senior author (JM). The extent of resection (EOR) was calculated using the formula 1 − [((Pre-Op Tm Volume – Post-Op Tm Volume) / Pre-Op Tm Volume) * 100]. In accordance with prior literature, EOR was independently determined by a board certified neuroradiologist (RKF) as: (1) gross total resection (i.e. GTR: ≥98% tumor removal), (2) near total resection (i.e. NTR: 90–98% tumor removal) or (3) subtotal resection (i.e. STR: <90% of tumor removal) [38]. For patients who were unable to undergo postoperative MRI, EOR was determined based on intraoperative imaging, post-operative CT and operative report.

### Intraoperative image guidance techniques

The use of the Brainlab Neuronavigation system and an operating microscope was standard in all surgeries. The 3D intraoperative ultrasound (IoUS) system (BK Flex Focus 800 or bk5000 Neurosurgical System) and 3T intraoperative magnetic resonance imaging (IoMRI) (3T MRI Scanner, Siemens MAGNETOM) were used as intraoperative image guidance modalities.

### Tumor pathology

Histological diagnosis of all tumors was determined by board-certified neuropathologists in accordance with WHO guidelines. MGMT status was determined in forty-seven patients (Table 1).

**Table 1:** General patient characteristics and comparative analysis between ioUS and combined ioMRI + ioUS groups

Characteristics	Total	IoUS	ioUS+IoMRI	P value
Age in Years (Mean ± SD)	75.98 ± 4.99	80.16 ± 5.99	74.43 ± 3.36	**0.0053**
Gender				
Female	20	3 (23.08%)	17	0.188
Male	28	10 (76.92%)	18	
Presentation				
Altered mental status	17 (35.42%)	5 (38.46%)	12 (34.29 %)	1
Facial strength	9 (18.75%)	2 (15.38%)	7 (20 %)	1
Language/Speech deficit				
Seizures	5 (10.42%)	2 (15.38%)	3 (8.57%)	0.602
Motor deficit and Ataxia	16 (33.33%)	6 (46.15%)	10 (28.57%)	0.311
Visual field cut	10 (20.83%)	1 (7.69%)	9 (25.71%)	0.248
Past medical history	44 (91.67%)	12 (92.31%)	32 (91.43%)	1
Hypertension	27 (56.25%)	7 (53.85%)	20 (57.14%)	1
Diabetes Mellitus	9 (18.75%)	1 (7.69%)	8 (22.86%)	0.411
Hyperlipidemia	23 (47.92%)	6 (46.1 %)	17 (48.57%)	1
Thyroid disorder	5 (10.42%)	0	5 (14.29%)	0.304
DVT/PE	4 (8.33%)	1 (7.69 %)	3 (8.57%)	1
* Cardiovascular Disorders*	17 (35.42 %)	8 (61.54 %)	9 (25.71%)	0.039
Arrhythmia	9 (18.75 %)	4 (30.77 %)	5 (14.29%)	0.228
Stroke	4 (8.33 %)	2 (15.38 %)	2 (5.71%)	0.294
CAD	4 (8.33%)	2 (15.38%)	2 (5.71%)	0.294
Aortic aneurysm	3 (6.25%)	3 (23.08%)	0	**0.017**
Aortic stenosis	2 (4.16%)	1 (7.69%)	1 (2.86%)	0.473
Localization				
Right	32 (66.67%)	7 (53.85%)	25 (71.43%)	0.311
Left	16 (33.33%)	6 (46.15%)	10 (28.57%)	
Anatomic classification				
Supratentorial	47 (97.92%)	13 (100%)	34 (97.14%)	1
Infratentorial	1 (2.08%)	0	1 (2.86%)	
Location				
Frontal	15 (31.25 %)	4 (30.77%)	11 (31.43%)	1
Temporal	22 (45.83 %)	6 (46.15%)	16 (45.71%)	1
Parietal	16 (33.33 %)	5 (38.46%)	11 (31.43%)	0.735
Occipital	3 (6.25%)	1 (7.69%)	2 (5.71%)	1
Multilobar	8 (16.67%)	3 (23.08%)	5 (14.29%)	0.665
Multifocal	1 (2.08 %)	1 (7.69%)	0	0.271
Eloquent structure involvement	16 (33.33 %)	5 (38.46%)	11 (31.43%)	0.735
Basal Ganglia	6 (12.50%)	3 (23.08%)	3 (8.57%)	0.323
Insula	8 (16.67%)	1 (7.69%)	7 (20%)	0.418
Thalamus	3 (6.25%)	2 (15.38%)	1 (2.86%)	0.265
Motor cortex	1 (2.08%)	1 (7.69%)	0	0.271
Large arterial encasement by the tumor	1 (2.08 %)	0	1 (2.86%)	1
Brainstem	1 (2.08%)	0	1 (2.86%)	1
Corpus callosum	0	0	0	NA
Median tumor volume	27.35	24.35	34.25	0.1454
MGMT status				
Methylated	17 (36.17%)	3 (6.38%)	14 (29.79%)	1*
Partially methylated	6 (12.77%)	3 (6.38%)	3 (6.38%)	
Non-Methylated	24 (51.06%)	6 (12.77%)	18 (36.30%)	

Bold values indicate statistical significance at the p < 0.05 level

*Methylated and partially methylated MGMT have been grouped together for the comparative analysis

*DVT* Deep venous thrombosis, *PE* Pulmonary embolus

### Statistical analysis

Statistical analyses were performed using SPSS (IBM SPSS Statistics, version 24) or Graphpad Prism version 8.3.0. Univariate analyses used either simple logistic regression model or one of the following: Fisher Exact test (categorical variables when two groups were compared); pairwise Fisher exact test (if more than two groups were compared); t-test with Welch correction (continuous variables with normal distribution); Mann Whitney-U test was conducted for any variables, which did not pass a Kolmogorov-Smirnov normality test; Wilcoxon Matched-Pairs Signed Rank test was used for paired variables with normal distribution. When the dependent variable was continuous, univariate and multivariate analyses were performed using linear regression models. For multivariate analysis, a binary logistic regression with a forward stepwise selection model was conducted using all variables tested in univariate analysis with an entry P value threshold 0.05 and removal threshold 0.1. Cox proportional hazards model was used to examine the impact of the variables on overall survival (OS). Kaplan Meier survival curves were performed to visualize OS for significant variables. P values < 0.05 were considered statistically significant.

## Results

### Cohort characteristics

Forty-seven patients had newly diagnosed GBM; one patient who presented with a “recurrent” GBM was operated on in another hospital 5 months prior to undergoing surgery with the senior author and had not received any adjuvant treatment. The average age was 75.98 years (range: 70–92). The median preoperative KPS score was 70 (range: 20–100), while the median KPS at 6-weeks follow-up was 90 (range: 20–100). The median tumor volume was 27.35 cm 3. These results are summarized in Tables 1 and 2.

**Table 2:** Descriptive analysis of the surgical outcome and comparison between the ioUS and combined ioMRI + ioUS group

Characteristics	Total	IoUS	ioUS+IoMRI	P value
Preoperative Karnofski performance scale (KPS) Score				
≥70	26 (54.17 %)	8 (61.54 %)	18 (51.43 %)	0.746
<70	22 (45.83 %)	5 (38.46 %)	17 (48.57 %)	
Preoperative NANO scale (median)	3	2.5	3	0.917
Extent of resection				
Gross total resection (GTR)	31 (64.58 %)	7 (53.84 %)	24 (68.58 %)	0.498
Near total resection (NTR)	12 (25 %)	3 (23.08 %)	9 (25.71 %)	1
Subtotal resection (STR)	5 (10.42 %)	3 (23.08 %)	2 (5.71 %)	0.115
Average length of surgery (minutes)	169.7 ± 58.93	111.1 ± 51.32	189.7 ± 47.11	**0.0002**
Postoperative complications *	8 (16.67 %)	1 (7.69 %)	7 (20 %)	0.418
Altered mental status	4 (8.33 %)	0	4 (11.43 %)	0.563
Urinary tract infection	3 (6.25 %)	0	3 (8.57 %)	0.553
Wound infection	2 (4.17 %)	0	2 (5.71 %)	1
Ileus	2 (4.17 %)	0	2 (5.71 %)	1
Postoperative hematoma (Managed without evacuation)	1 (2.08 %)	1 (7.69 %)	0	0.271
Pulmonary embolus	1 (2.08 %)	0	1 (2.86 %)	1
Perforator infarct	1 (2.08 %)	0	1 (2.86 %)	1
Atrial fibrillation	1 (2.08 %)	0	1 (2.86 %)	1
Average length of the hospital stay after the surgery (Days)	4.583 ± 3.451	4.769 ± 2.891	4.514 ± 3.673	0.803
Disposition				
Home	23 (47.92 %)	6 (46.15 %)	17 (48.57 %)	1
Skilled nursing facilities (Short-term)	16 (33.33 %)	5 (38.46 %)	11 (31.43 %)	0.735
Rehabilitation centers	9 (18.75 %)	2 (15.38 %)	7 (20 %)	1
Functional outcome (Median)				
Postoperative KPS score (at discharge)	**70**	70	70	0.964
Postoperative follow-up KPS score (at 6 weeks follow-up)	90	80	90	0.382
Median Follow-up NANO scale	1	1	1	0.722
Changes in functional outcome				
Improvement	28 (58.33 %)	6 (46.15 %)	22 (62.86 %)	0.339
Unchanged	12 (25 %)	5 (38.46 %)	7 (20 %)	0.263
Worsening	8 (16.67 %)	2 (15.38 %)	6 (17.14 %)	1
Chemoradiation protocols				
Stupp protocol	24 (50 %)	4 (30.77 %)	20 (57.14 %)	0.193
Perry protocol	5 (10.42 %)	0	5 (14.29 %)	0.304
Hypofractionated radiation treatment alone	8 (16.67 %)	4 (30.77 %)	4 (11.43 %)	0.187
No therapy	11 (22.92 %)	5 (38.46 %)	6 (17.14 %)	0.14

Bold values indicate statistical significance at the p < 0.05 level

*Some patients developed more than one complication

GTR or NTR were achieved in forty-three cases (89.58%). Average length of hospital stay was 4.58 days, with 47.92% of patients being discharged home. Twenty-four patients (50%) received adjuvant treatment according to Stupp Protocol [39], five patients (10.42%) according to Perry Protocol [3] and eight patients (16.67%) received hypo-fractionated radiotherapy alone. Eleven patients (22.92 %) did not receive any adjuvant treatment.

### Comparison of intraoperative imaging modalities

A combination of IoMRI and IoUS was used in 35 cases, while IoUS solely was used in 13 cases. There were no statistically significant differences between the IoUS and IoUS+IoMRI groups in terms of gender (Fisher Exact test, P = 0.188), pre-operative tumor volume (Mann-Whitney test, P = 0.15), involvement of eloquent structures (Fisher Exact Test; P = 0.74), MGMT status (Fisher Exact test, P = 1) or preoperative KPS score (Mann-Whitney test, P = 0.96). Patients in the IoUS group, however, were significantly older (t-test with Welch correction, P = 0.005) (Table 1).

While there was no difference in EOR (Pairwise Fisher Exact test, P = 0.263) between the groups, the LOSx was significantly longer (t-test with Welch correction, P = 0.0002) in the IoMRI group. There were no significant differences however with regards to postoperative complications (Fisher Exact test, P = 0.42), length of hospital stay (Mann-Whitney test, P = 0.80) or place of disposition (Pairwise Fisher Exact Test, P = 0.918). There were no differences in terms of number of patients who received adjuvant treatment with Stupp Protocol [39] (Fisher Exact test, P = 0.193), Perry Protocol [3] (Fisher Exact test, P = 0.304), hypo-fractionated radiotherapy alone (Fisher Exact test, P = 0.187) or who did not receive any adjuvant treatment (Fisher Exact test, P = 0.14) (Table 2). Patients in both groups showed significant functional improvement at 6 weeks postoperative follow-up (Wilcoxon matched-pairs signed Rank Test, P = 0.039 and P = 0.0003 in ioUS and ioMRI+ioUS, respectively).

### Postoperative complications

Postoperative complications were observed in 8 patients (16.67%), some of whom experienced more than one issue. Several of these complications were related to medical care, including urinary tract infections and pulmonary embolus (Table 2). Prolonged surgery time (OR = 1.023, 95%, CI 1.005–1.043; P = 0.013), pre-operative KPS score less than 70 (OR = 11.67, 95% CI 1.305–104.337; P = 0.028) and prolonged hospital stay (OR = 1.249, 95% CI 1.025–1.522; P = 0.028) were among the significant predictors of complications.

The frequency of postoperative complications in the ioMRI+ioUS group was more than two-times as much as the ioUS-alone cases (20–7.69%), though this difference was not statistically significant (Fisher Exact Test, P = 0.42).

LOSx (OR = 1.033, 95% CI 1.006–1.06; P = 0.015) and hospital stay (OR = 1.422, 95% CI 1.046–1.932; P = 0.025) remained significant in multiple logistic regression analysis (Table 3), suggesting that longer time spent in surgery and the hospital increases the likelihood of postoperative complications.

**Table 3:** Logistic regression analysis of postoperative complications

Characteristics	Odds Ratio (95% CI)	P value
*Univariate analysis simple logistic regression*		
Length of surgery	1.023 (1.005–1.043)	**0.013**
Pre-operative KPS (KPS score ≥70 vs. KPS score <70)	0.085 (0.010–0.767)	**0.028**
Length of hospital stay	1.249 (1.025–1.522)	**0.028**
Tumor volume	0.974 (0.934–1.016)	0.226
Pre-operative language deficit	2.4 (0.471–12.22)	0.292
Gender	0.36 (0.075–1.727)	0.202
Image guidance technique (ioUS vs. ioMRI + ioUS)	0.333 (0.037–3.014)	0.328
Pre-operative motor deficit	1.246 (0.258–6.031)	0.784
Age	0.961 (0.810–1.139)	0.644
Pre-operative altered mental status	0.556 (0.099–3.113)	0.504
Involvement of eloquent structures	1.246 (0.257–6.031)	0.784
*Stepwise-Forward Multiple Logistic Regression*		
Length of surgery	1.033 (1.006–1.06 )	**0.015**
Length of hospital stay	1.422 (1.046 –1.932)	**0.025**

Bold values indicate statistical significance at the p < 0.05 level

* KPS* Karnofski Performance Scale; ioUS, intraoperative ultrasonography, *ioMRI* intraoperative magnetic resonance imaging. NANO, The Neurologic Assessment in Neuro-Oncology

### Discharge disposition

We analyzed the place of disposition as a reflection of immediate postoperative functional outcome. 23 patients (47.92 %) were discharged home, while 16 patients (33.33%) were discharged to short-term skilled nursing facilities (SNF) and 9 (18.75 %) to rehabilitation centers. The multiple logistic regression model demonstrated that patients with a preoperative KPS score less than 70 (OR = 0.211, 95% CI 0.061–0.732; P = 0.014) were less likely to be discharged home and more likely to SNF or rehabilitations centers.

### Follow-up KPS score at postoperative six week and functional improvement

We next sought to elucidate the variables contributing to the follow-up KPS score at postoperative 6 weeks. Univariate analysis implicated postoperative complications (OR = 0.15, 95% CI 0.029–0.764; P = 0.022), and preoperative language deficit (OR = 0.194, 95 % CI 0.046–0.824; P = 0.026) as significant predictors of follow-up KPS score. In multivariate logistic regression model, postoperative complications (OR = 0.161, 95% CI 0.03- 0.904; P = 0.038) and preoperative language deficit (OR = 0.208, 95% CI 0.045–0.971; P = 0.046) remained significant to predict a follow-up KPS score less than 70 at postoperative 6 weeks.

**Fig. 1:**
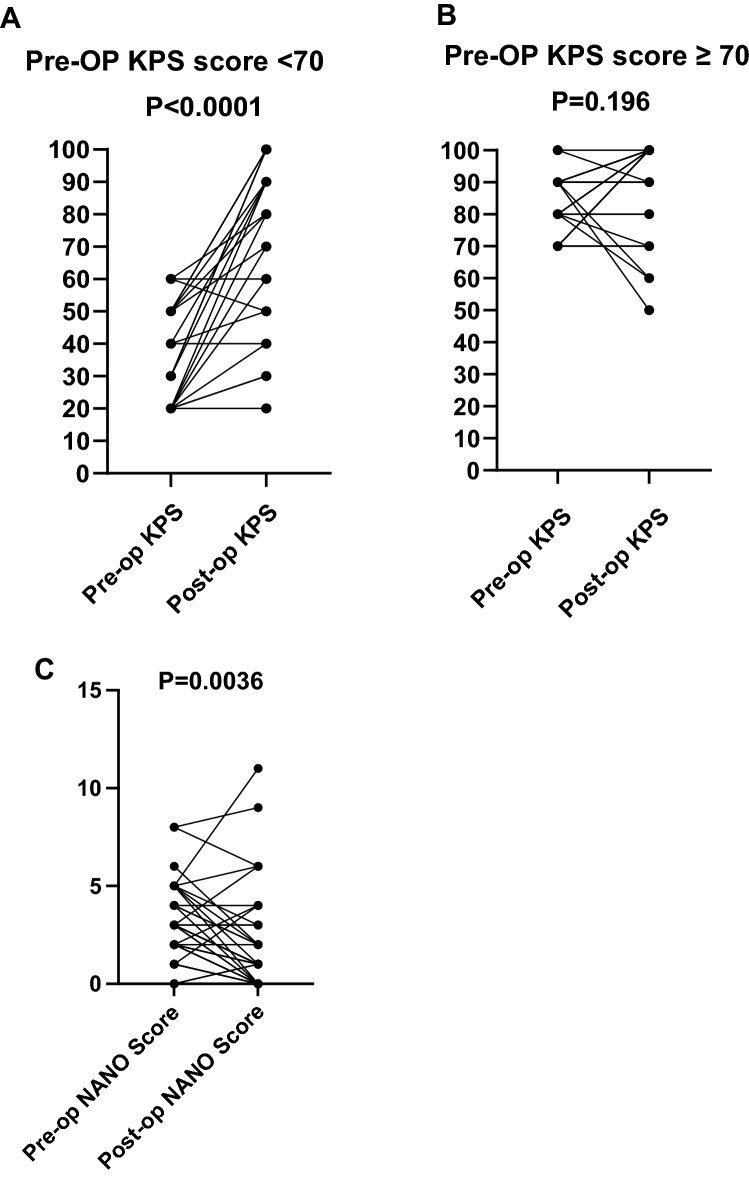
Patients with pre-operative KPS score <70 demonstrated significant improvement 6-weeks after surgical resection. Paired parallel axis dot plot showing significant functional improvement for patients with pre-op KPS score <70 following the surgical resection (p<0.0001) (**A**) ,while no significant changes were observed in pre-op KPS score ≥ 70 group (**B**)**.** Preoperative NANO scale improved significantly at postoperative follow-up assessment (P = 0.0036) from a preoperative median score of 3 to 1 at follow-up (**C**). Abbreviations: Pre-op, preoperative; post-op, postoperative; KPS, Karnofski Performance Scale; NANO, The Neurologic Assessment in Neuro-Oncology

We further analyzed the factors associated with postoperative functional improvement, which we defined as an at least 10-point difference between the follow-up KPS and the preoperative KPS scores. We found that 28 patients (58.33%) showed functional improvement, while 12 patients (25%) remained the same and 8 patients (16.67%) worsened. Interestingly, 59% of patients with a preoperative KPS score less than 70 showed a follow-up KPS score 70 or higher and showed significant functional improvement (Wilcoxon matched-pairs signed Rank Test, P<0.0001), although patients with a preoperative KPS score 70 or higher did not show a significant change (P = 0.196) (Fig. 1). This result remained the same (P = 0.1578) when we excluded two patients with a preoperative KPS score of 100 from the analysis. Taken together, these results suggest that patients with relatively lower preoperative KPS scores can demonstrate significant functional improvement following surgery, while those with higher scores maintain them.

Forty-three patients, who had clear physical examination records necessary for all components of scoring were assessed using the NANO scale. Median preoperative and follow-up NANO scale scores were 3 and 1, respectively. A significant improvement was observed when the functional status of patients was evaluated using NANO scale (P = 0.0036, Wilcoxon Matched-Pairs Signed Rank test) (Fig. 1). 28 (65%) of the patients showed at least 1 level improvement in NANO scale, while 20 patients (46.5%) showed at least 2 levels, and 10 (23.3%) patients showed at least 3 levels improvement.

### Postoperative adjuvant treatment

We next elucidated variables contributing to the likelihood patients went on to receive postoperative adjuvant treatment, including the Stupp protocol [39], Perry protocol [3] or hypofractionated RT only.

We found that a preoperative KPS score 70 or higher showed the most significant association in univariate analysis (OR = 8.307, 95% CI 1.557–44.32; P = 0.013) with more likelihood to proceed with adjuvant therapy, which was followed by the KPS score at postoperative 6 weeks (OR = 5.143, 95% CI 1.214–21.795; P = 0.026).

Preoperative KPS score was significantly associated with postoperative adjuvant treatment in multivariate analysis (OR = 16.251, 95% CI 2.048–128.944; P = 0.008). However, when we adjusted the analysis for the postoperative KPS score, we found that the preoperative KPS score is not a significant predictor of postoperative adjuvant treatment for the patients who had a follow-up KPS score less than 70 (P = 0.99), underscoring the importance of postoperative KPS score in decision-making for postoperative adjuvant treatment.

These findings suggest that the functional status of older GBM patients after surgery is an important determinant in whether patients are able to continue with postoperative adjuvant treatment, and not necessarily predictive pre-operatively.

### Survival analyses

Two patients were excluded: One who was operated in another institution for GBM five months prior to the second surgery in our institution was excluded, and another with COVID19 during the radiation treatment, which resulted in interruption of the treatment and death soon thereafter.

Univariate analysis showed the presence of postoperative complications (HR = 4.119, 95% CI 1.753–9.679; P = 0.001) was predictive for overall survival (OS). In this cohort, we found that postoperative adjuvant therapy (any aforementioned type) is associated with improved OS compared to none (HR = 0.075, 95% CI 0.028–0.204, P<0.0001).

In multivariate analysis, we found that greater EOR (GTR or NTR vs. STR) (Median OS = 281 vs. 163 days; HR = 0.329, 95% CI 0.121–0.896; P = 0.030) and use of postoperative adjuvant treatment (Median OS = 335 vs. 110; HR = 0.144, 95% CI 0.057 – 0.362; P<0.0001), are associated with improved OS in this population of patients. Overall survival, however, was significantly shorter with the presence of postoperative complications (Median OS = 120 vs. 305 days; HR = 3.620, 95% CI 1.433 – 9.144, P = 0.006) (Table 4).

**Table 4:** Univariate and Multivariate Analysis of overall survival with Cox proportional hazards model

Characteristics	Hazard ratio (95% CI)	P value
*Univariate analysis*		
Adjuvant treatment	0.075 (0.028–0.204)	**3.47e–7**
Post-operative complications	4.119 (1.753–9.679)	**0.001**
Pre-operative KPS (KPS score ≥70 vs. KPS score <70)	0.559 (0.301–1.040)	0.066
EOR (GTR+NTR vs. STR)	0.540 (0.209–1.400)	0.205
MGMT * (methylated and partially methylated vs. unmethylated)	0.694 (0.366–1.317)	0.264
Gender	1.431 (0.762–2.690)	0.265
Image guidance technique (ioUS vs. ioMRI + ioUS)	1.450 (0.712–2.954)	0.306
Pre-operative altered mental status	1.290 (0.677–2.460)	0.439
Involvement of eloquent structures	1.192 (0.621–2.289)	0.598
Pre-operative motor deficit	1.175 (0.606–2.281)	0.633
Tumor volume	0.998 (0.990–1.008)	0.802
Follow-Up KPS (KPS score ≥70 vs. KPS score <70)	1.044 (0.495–2.203)	0.910
*Multivariate analysis*		
EOR (GTR+NTR vs. STR)	0.329 (0.121–0.896)	**0.030**
Post-operative complications	3.620 (1.433–9.144)	**0.006**
Adjuvant treatment	0.144 (0.057–0.362)	**3.80e–5**

Bold values indicate statistical significance at the p < 0.05 level

*An independent univariate analysis was further performed to assess the impact of MGMT status on overall survival in patients who had MGMT status determined as “methylated” or “unmethylated” and received temozolomide treatment (n = 29), (**P = 0.172**, HR:0.550, 95% CI 0.233 – 1.30)

Abbreviations: KPS, Karnofski Performance Scale; ioUS, intraoperative ultrasonography; ioMRI, intraoperative magnetic resonance imaging; EOR, Extent of resection; GTR, gross total resection; NTR, near total resection; STR, subtotal resection

## Discussion

GBM remains a challenging disease with an overall poor prognosis despite the most aggressive, multimodal treatment. Surgery, followed by chemoradiation, can be quite demanding, particularly for older individuals, therefore raising the question of how aggressive to be. Paradigms have been modified with regards to other aspects of GBM treatment in this population [3, 39], and thus we sought to determine whether surgical strategies can be similarly optimized.

In support of previous reports [4–6, 12–19], we demonstrated that older patients with GBM who undergo maximal EOR and adjuvant chemoradiation have an improved OS, suggesting that surgical resection and postoperative adjuvant treatment should not be withheld from this patient population. Patients with relatively lower pre-op KPS scores (<70) were more likely to be discharged to short-term facilities but showed significant improvement at postoperative 6-weeks. In fact, 59% of these patients received a follow-up KPS score of 70 or more at that time, which was a prognostic threshold important for predicting whether patients received postoperative adjuvant treatment. Indeed, patients who received postoperative chemoradiation, tolerated the treatment well and demonstrated improved OS compared to patients who did not receive adjuvant treatment. Thus, older patients, even those with relatively low preoperative KPS, benefit from surgery and can improve from their baseline preoperative functional state to tolerate adjuvant treatment and reap the OS benefits. Survival and quality of life outcomes, however, were significantly impacted by the presence of preoperative language deficit and postoperative complications, the latter of which was predictive for poor OS and follow-up KPS score at postoperative 6-weeks.

Given the prognostic implications of postoperative complications, and the trend that they increase with longer surgical time, surgical techniques and intraoperative adjuncts should be carefully considered to minimize complication risk, while aiming for maximal EOR. Similar to others, when investigating the usefulness of the addition of ioMRI, we found it does not offer any advantage for OS or functional improvement in this population. Indeed, a recent meta-analysis compared IoUS, IoMRI and other image guidance techniques in high grade glioma surgery and similarly found no significant difference between these modalities in terms of EOR and survival, while ioMRI use was significantly more expensive [31]. However, these studies did not compare clinical outcome in older patients. Indeed, ioMRI use in older patients in our cohort, significantly prolonged surgery, which served as a risk factor for postoperative complications in these patients, which were predictive of follow-up KPS score at postoperative 6-weeks and poor overall survival. There was a trend toward an increased frequency of postoperative complications in combined ioMRI+ioUS group compared to ioUS-alone cases (20–7.69%) despite the latter group being older on average. Though this difference did not reach statistical significance, possibly due to sample size, this finding suggests that surgery in older patients with GBM should be aimed at minimizing the operative time and avoiding the use of unnecessary adjuncts. In experienced hands, ioUS can garner similar benefits as IoMRI, while potentially avoiding deleterious ones. While this is the strategy now employed by the senior author, it should be noted that older patients should be assessed on a case-by-case basis such that the addition of ioMRI could be justified in certain patients and circumstances.

Our study has limitations. First, it is retrospective and of a single center designed with a homogenous, but relatively small patient cohort. This could explain why no survival benefit with regards to MGMT status or pre-op KPS was observed, as well as the small number of those patients undergoing STR. Second, as expected, a significant association was found between EOR and improved OS in multivariate analysis, but oddly not observed in univariate analysis. While this may seem counterintuitive, other variables that impact OS (e.g., complications) may act as negative confounders for the association between GTR and OS in multivariate analysis, rendering the significance of EOR in multivariate, but not univariate analysis. As described before, the inclusion of negative confounders in the model would push the adjusted association away from the null, leading to the significance of EOR more significant in multivariate analysis [40]. Additionally, it is important to note that the average age of the patients in ioUS group was significantly older than the combined group. This difference was due to the senior’s author intention to reduce the duration of surgeries in this relatively more senior age group, based on our hypothesis. However, the relatively older age in the ioUS group, would render that group at a disadvantage from a prognostic standpoint, which further underscores the observation. This study can serve as a pilot for data building the reasoning for further prospective studies with larger study cohorts.

## Conclusions

Aggressive surgical resection improves OS and functional outcomes in older patients with GBM, and therefore maximal interventions should be considered in this patient population. In experienced hands, ioMRI use does not add any significant benefit over ioUS in terms of OS and functional outcomes in older GBM patients, but does increase the length of surgery, a preventable prognostic factor for postoperative complications.

## Supplementary Information

Below is the link to the electronic supplementary material.

Supplementary material 1 (DOCX 573.2 kb)

Supplementary material 2 (DOCX 542.1 kb)
