# Correction to: Surgical strategies for older patients with glioblastoma

**DOI:** 10.1007/s11060-021-03888-3

**Published:** 2021-11-23

**Authors:** Tanyeri Barak, Shaurey Vetsa, Arushii Nadar, Lan Jin, Trisha P. Gupte, Elena I. Fomchenko, Danielle F. Miyagishima, Kanat Yalcin, Sagar Vasandani, Evan Gorelick, Amy Y. Zhao, Joseph Antonios, Brianna Carusillo Theriault, Nathan Lifton, Neelan Marianayagam, Bulent Omay, Zeynep Erson Omay, Anita Huttner, Declan McGuone, Nicholas A. Blondin, Zachary Corbin, Robert K. Fulbright, Jennifer Moliterno

**Affiliations:** 1 Department of Neurosurgery, Yale School of Medicine, 15 York St., LLCI 810, CT 06520-8082 New Haven, USA; 2 Yale Brain Tumor Center, Smilow Cancer Hospital, CT New Haven, USA; 3 Department of Surgery, Yale School of Medicine, New Haven, USA; 4 Department of Environmental Health Sciences, Yale School of Public Health, New Haven, USA; 5 Department of Pathology, Yale School of Medicine, New Haven, USA; 6 Department of Neurology, Yale School of Medicine, New Haven, USA; 7 Department of Radiology and Biomedical Imaging, Yale School of Medicine, New Haven, USA; 8 Department of Genetics, Yale School of Medicine, New Haven, Connecticut, USA

## Correction to: Journal of Neuro-Oncology 10.1007/s11060-021-03862-z

The article *Surgical strategies for older patients with glioblastoma*, written by Tanyeri Barak, Shaurey Vetsa, Arushii Nadar, Lan Jin, Trisha P. Gupte, Elena I. Fomchenko, Danielle F. Miyagishima, Kanat Yalcin, Sagar Vasandani, Evan Gorelick, Amy Y. Zhao, Joseph Antonios, Brianna Carusillo Theriault, Nathan Lifton, Neelan Marianayagam, Bulent Omay, Zeynep Erson Omay, Anita Huttner, Declan McGuone, Nicholas A. Blondin, Zachary Corbin, Robert K. Fulbright & Jennifer Moliterno, was originally published electronically on the publisher’s internet portal on 9 October 2021 without open access. With the author(s)’ decision to opt for Open Choice the copyright of the article changed on 27 October 2021 to © The Author(s) 2021 and the article is forthwith distributed under a Creative Commons Attribution.

**Open Access** This article is licensed under a Creative Commons Attribution 4.0 International License, which permits use, sharing, adaptation, distribution and reproduction in any medium or format, as long as you give appropriate credit to the original author(s) and the source, provide a link to the Creative Commons licence, and indicate if changes were made. The images or other third party material in this article are included in the article’s Creative Commons licence, unless indicated otherwise in a credit line to the material. If material is not included in the article’s Creative Commons licence and your intended use is not permitted by statutory regulation or exceeds the permitted use, you will need to obtain permission directly from the copyright holder. To view a copy of this licence, visit http://creativecommons.org/licenses/by/4.0/.

