# Supplementary material for: Surgical strategies for older patients with glioblastoma
Source: J Neurooncol. 2021 Oct 9;155(3):255–64. doi: 10.1007/s11060-021-03862-z (PMC8651607; doi:10.1007/s11060-021-03862-z)
Supplement: Supplementary file 1 — Supplementary material 1 (DOCX 573.2 kb) [file 11060_2021_3862_MOESM1_ESM.docx]

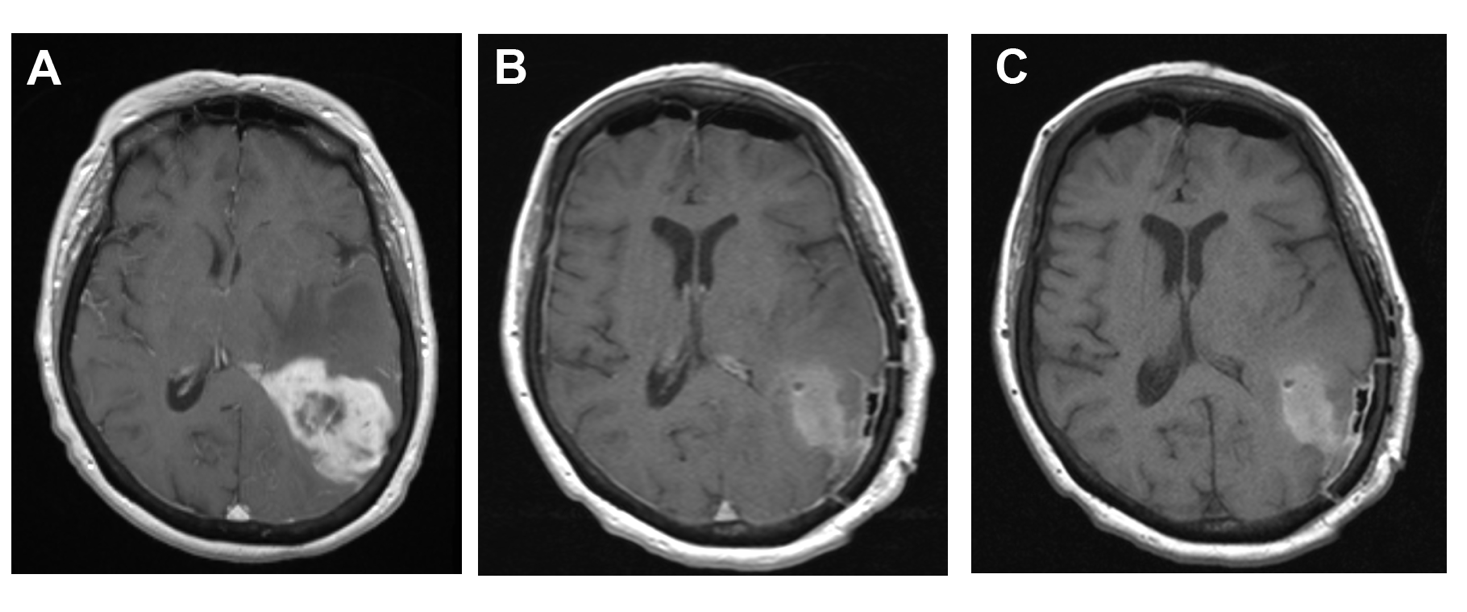
Appendix 1

A 71 year-old female with no significant past medical history presented with aphasia and unsteady gait and found to have a large left temporal heterogeneously enhancing intra-axial mass, shown in preoperative post-gadolinium T1-weighted image in A. Her KPS was 50 and NANO scale score was 6 on admission. She underwent gross total resection of the tumor, demonstrated with postoperative post-gadolinium T1-weighted image in B and pre-gadolinium T1-weighted image in C (hyperintensity in B and C is blood). Intraoperative ultrasonography (ioUS) was used for intraoperative imaging and neuronavigation. The pathology confirmed the diagnosis of glioblastoma multiforme. The tumor was IDH wildtype and MGMT promoter was unmethylated. Her post-operative course was uneventful and she was discharged home on post-operative day 4. Her KPS at discharge was 70 and at six weeks follow up, her KPS was 90, while NANO scale score was 2.
