# Supplementary material for: Surgical strategies for older patients with glioblastoma
Source: J Neurooncol. 2021 Oct 9;155(3):255–64. doi: 10.1007/s11060-021-03862-z (PMC8651607; doi:10.1007/s11060-021-03862-z)
Supplement: Supplementary file 2 — Supplementary material 2 (DOCX 542.1 kb) [file 11060_2021_3862_MOESM2_ESM.docx]

Appendix 2


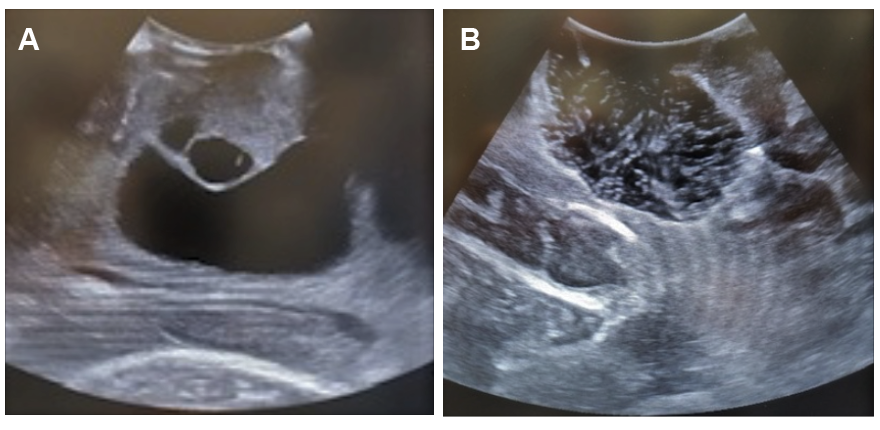


Example of the use of intraoperative ultrasonography (ioUS) to obtain gross total resection of a tumor, that was confirmed as glioblastoma multiforme by pathology. **A**, the cystic and solid components of the lesion before the resection. **B**, complete removal of the lesion using ioUS, surgical cavity filled with water.
